# Localization, induction, and functional effects of tau phosphorylated at threonine 217

**DOI:** 10.1002/alz.089421

**Published:** 2025-01-03

**Authors:** George S Bloom

**Affiliations:** ^1^ University of Virginia, Charlottesville, VA USA

## Abstract

**Background:**

Tau phosphorylated at threonine‐217 (tau_pT217_), which can be measured in plasma and CSF using antibodies, is one of the most promising early biomarkers for Alzheimer’s disease (AD). Little was known, however, about the cellular and subcellular distributions of tau_pT217_, how those levels change during disease progression, the factor(s) that provoke tau_pT217_ accumulation, or functional consequences of tau phosphorylation at T217. This study addressed all of those issues.

**Method:**

We defined the tau_pT217_ distribution *in vivo* by surveying brains of human AD patients at various disease stages and in age‐matched, cognitively normal controls, in AD model mouse brains, and *in vitro* in cultured mouse neurons. We also used cultured neurons to determine if extracellular oligomers of amyloid‐β (xcAβOs) or tau (xcTauOs) affect the abundance or localization of tau_pT217_. Finally, to determine if tau phosphorylation at T217 affects its affinity for microtubules, we expressed EGFP‐tagged wild type (WT) and pseudo‐phosphorylated tau in fibroblasts, and used fluorescence recovery after photobleaching to measure turnover rates of microtubule‐bound tau.

**Result:**

We first discovered that the tau_pT217_ epitope is found in 19 human proteins, but that specific and exclusive antibody recognition of tau_pT217_ can be achieved when suitable precautions are taken, We next determined that: 1) tau_pT217_ appears in neurons at Braak stages I‐II, and becomes more prevalent at later disease stages in axons, dendrites, neuronal cell bodies, neuropil threads, pre‐tangles and tangles that are extensively co‐labeled by the AT8 (tau_pS202/pT205_) and PHF1 (tau_pS396/pS404_) antibodies; 2) in cultured neurons, tau_pT217_ is increased by xcTauOs, but not by xcAβOs, and is associated with developing post‐synaptic sites; and 3) pseudo‐phosphorylation by a T217E amino acid substitution reduces tau’s affinity for microtubules.

**Conclusion:**

Early AD biomarker assays based on tau_pT217_ detection with antibodies are at risk of artifactual signal inflation unless rigorous steps are taken to ensure antibody specificity. Neuronal levels of tau_pT217_ rise steadily in brain as AD progresses. Finally, our collective results raise the possibilities that xcTauOs drive T217 phosphorylation of tau *in vivo*, and that tau_pT217_ contributes to synaptic decline and favors formation of tau‐tau interactions at the expense of tau binding to microtubules.

